# Denitrification in low oxic environments increases the accumulation of nitrogen oxide intermediates and modulates the evolutionary potential of microbial populations

**DOI:** 10.1111/1758-2229.13221

**Published:** 2023-11-30

**Authors:** Kohei Takahashi, Mamoru Oshiki, Chujin Ruan, Kana Morinaga, Masanori Toyofuku, Nobuhiko Nomura, David R. Johnson

**Affiliations:** ^1^ Graduate School of Sciences and Technologies University of Tsukuba Tsukuba Ibaraki Japan; ^2^ Department of Environmental Microbiology Swiss Federal Institute of Aquatic Science and Technology (Eawag) Dübendorf Switzerland; ^3^ Division of Environmental Engineering, Faculty of Engineering Hokkaido University Sapporo Hokkaido Japan; ^4^ Faculty of Life and Environmental Sciences University of Tsukuba Tsukuba Ibaraki Japan; ^5^ Microbiology Research Center for Sustainability University of Tsukuba Tsukuba Ibaraki Japan; ^6^ Institute of Ecology and Evolution University of Bern Bern Switzerland

## Abstract

Denitrification in oxic environments occurs when a microorganism uses nitrogen oxides as terminal electron acceptors even though oxygen is available. While this phenomenon is well‐established, its consequences on ecological and evolutionary processes remain poorly understood. We hypothesize here that denitrification in oxic environments can modify the accumulation profiles of nitrogen oxide intermediates with cascading effects on the evolutionary potentials of denitrifying microorganisms. To test this, we performed laboratory experiments with *Paracoccus denitrificans* and complemented them with individual‐based computational modelling. We found that denitrification in low oxic environments significantly increases the accumulation of nitrite and nitric oxide. We further found that the increased accumulation of these intermediates has a negative effect on growth at low pH. Finally, we found that the increased negative effect at low pH increases the number of individuals that contribute to surface‐associated growth. This increases the amount of genetic diversity that is preserved from the initial population, thus increasing the number of genetic targets for natural selection to act upon and resulting in higher evolutionary potentials. Together, our data highlight that denitrification in low oxic environments can affect the ecological processes and evolutionary potentials of denitrifying microorganisms by modifying the accumulation of nitrogen oxide intermediates.

## Graphical abstract

We show that denitrification in low oxic environments increases the accumulation of nitrite and nitric oxide intermediates. At low pH, the toxicity of these intermediates reduces the rate of growth and increases the number of individuals that contribute to surface‐associated growth. This increases the amount of genetic diversity that is preserved during surface‐associated growth, thus increasing the number of genetic targets for natural selection to act upon and resulting in higher evolutionary potentials.

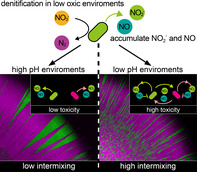

## INTRODUCTION

Denitrifying microorganisms, which sequentially reduce nitrate, nitrite, nitric oxide and nitrous oxide in a respiratory process, have a central role in the biogeochemical cycling of nitrogen by transforming nitrogen oxides into inert nitrogen gas (Stein & Klotz, 2016; Zumft, 1997). A key feature of this pathway is that nitrogen oxide intermediates often accumulate during the process (nitrite, nitric oxide and nitrous oxide) with potentially deleterious effects on ecosystems (Almeida et al., 1995; Frostegård et al., 2021; Pan et al., 2015; Philips et al., 2002). For example, nitrite and nitric oxide can have severe toxic effects in low pH environments. Nitrite will protonate into free nitrous acid, which has strong biocidal effects on many microorganisms (Jiang et al., 2011; Jin et al., 2012; Wang et al., 2023; Zhou et al., 2011). Moreover, nitric oxide can directly disrupt respiration systems and interact with superoxide to form peroxynitrite, which in turn can oxidize lipids, proteins and nucleic acids (Fang, 2004; Pacher et al., 2007). Finally, nitrous oxide is not only a major greenhouse gas and ozone‐depleting substance (Ravishankara et al., 2009; Stein & Klotz, 2016), but can also inhibit key microbial processes important for environmental quality and sustainability (Yin et al., 2019). Taken together, it is clear that the accumulation profiles of nitrogen oxide intermediate during denitrification can have significant effects on ecosystem functioning across a variety of spatial scales, and a comprehensive understanding of the causes of intermediate accumulation and its impact on microbial communities is therefore vital for predicting, maintaining and controlling the nitrogen cycle in both natural and engineered ecological systems.

In addition to ecological processes, the accumulation of nitrogen oxide intermediates can also modify the evolutionary processes acting on denitrifying populations. For example, the transient accumulation of nitrite by a completely denitrifying microorganism, which is widely observed among environmental isolates (Betlach & Tiedje, 1981; Lilja & Johnson, 2016), can create a niche opportunity for a partial denitrifying microorganism that specializes at reducing nitrite (Dolinšek et al., 2022). Stated more generally, the accumulation of nitrogen oxide intermediates (or more generally any intermediate) can create new habitats and niches for metabolically specialized microorganisms to occupy, thus enabling the emergence and maintenance of microbial diversity (Dolinšek et al., 2022; Johnson et al., 2012). In another study, Lilja and Johnson modified nitrite toxicity by controlling the pH and quantified the effect on the pace of molecular evolution (Lilja & Johnson, 2017). They found that increasing nitrite toxicity increases the number of available beneficial mutations that selection can act upon, thus accelerating the pace of molecular evolution (Lilja & Johnson, 2017). Thus, the accumulation of nitrogen oxide intermediates can have important effects on the life histories of denitrifying microorganisms.

While the ecological and evolutionary consequences of accumulating nitrogen oxide intermediates during denitrification are becoming apparent, our understanding of the causes and consequences of nitrogen oxide accumulation are largely limited to denitrification in anoxic environments. However, there are many microorganisms that can perform denitrification in oxic environments (Ji et al., 2015; Song et al., 2021; Yang et al., 2020). Denitrification in oxic environments refers to the ability of some microorganisms to respire nitrogen oxides in the presence of oxygen or to co‐respire nitrogen oxides and oxygen simultaneously (Li et al., 2021; Zhang et al., 2018; Zhang et al., 2020). Importantly, denitrification in oxic environments can have implications for nitrogen cycling and environmental management. However, the ecological and evolutionary consequences of nitrogen oxide accumulation during denitrification in oxic environments remain poorly understood. A deeper understanding of these consequences could have practical implications for water and soil quality management, such as managing nitrogen loads in wastewater treatment processes and agriculture. In addition, microorganisms capable of denitrification in oxic environments are being exploited as agents for bioremediation of contaminated sites and as producers of bioenergy and other value‐added products (Li et al., 2020; Pai et al., 1999).

In this study, we hypothesize that denitrification in oxic environments can modify the accumulation profiles of nitrogen oxide intermediates with cascading effects on the growth and evolutionary potentials of denitrifying populations. To test this hypothesis, we performed laboratory experiments with *Paracoccus denitrificans*, which is a model bacterium for studying denitrification (Baumann et al., 1996; Medhi et al., 2017; Zhang et al., 2020). Our goal was to quantitatively compare the accumulation profiles of nitrogen oxide intermediates in oxic and anoxic environments and then measure the consequences on both liquid and surface‐associated growth. We performed further experiments to test how the accumulation of nitrogen oxide intermediates affects the maintenance of genetic diversity during surface‐associated growth, and thus how they may affect the evolutionary potentials of *P. denitrificans* populations. Finally, we used an individual‐based computational model to identify an ecological mechanism for how nitrogen oxide intermediates could affect evolutionary potentials. Together, our research aims to shed light on the ecological and evolutionary consequences of denitrification in oxic environments, and thus improve our ability to predict and control denitrification processes.

## EXPERIMENTAL PROCEDURES

### Bacterial strains and growth conditions

We provide a list of all the bacterial strains and plasmids used in this study in Table S1. We routinely grew all *P. denitrificans* strains on lysogeny broth (LB) agar plates or in typtic soy broth (TSB) liquid medium. We routinely grew all *Escherichia coli* strains on LB agar plates or in LB liquid medium. We incubated all *P. denitrificans* cultures at 30°C and all *E. coli* cultures at 37°C. When necessary to maintain one of the plasmids listed in Table S1, we supplemented the medium with 50 μg mL^−1^ kanamycin.

### Construction of derivative strains

To construct the enhanced green fluorescent protein‐based promoter‐reporter derivative strains of *P. denitrificans* PD1222, we engineered an mNeonGreen fluorescent protein to achieve high brightness with optimized codon usage for bacteria (Table S2). For construction of the promoter‐reporter, we first PCR‐amplified the *mNeonGreen* gene with primers mNeon‐Cp_F and mNeon‐Cp_R (Table S3) and cloned the amplicons into the pPROBE‐NT vector by InFusion cloning (Takara Bio, Kusatsu, Japan) to construct the plasmid pPROBE‐NT‐mNeonGreen (Table S1). We next PCR‐amplified the promoter regions located immediately upstream of the following genes using the primer sets listed in Table S3; Pden_4237 (*narK*) (genomic positions 1,403,768–1,404,041), Pden_2487 (*nirS*) (genomic positions 250,272–250,399), Pden_2486 (*nirI*) (genomic positions 250,272–250,399) and Pden_4219 (*nosZ*) (genomic positions 1,385,204–1,385,540). Each promoter is located upstream of genes that encode for essential components of the nitrate, nitrite, nitric oxide or nitrous oxide reductase, respectively (Li et al., 2020; Pai et al., 1999). We then cloned the PCR‐amplified promoter sequences upstream of the *mNeonGreen* gene located in the pPROBE‐mNeonGreen‐NT vector by InFusion cloning (Takara Bio, Kusatsu, Japan). Finally, we introduced the resulting plasmids, referred to as pRPOBE‐NT‐Pnar‐mNeonGreen, pRPOBE‐NT‐Pnir‐mNeonGreen, pRPOBE‐NT‐Pnor‐mNeonGreen and pRPOBE‐NT‐Pnos‐mNeonGreen (Table S1), into strain PD1222 by conjugation from *E. coli* S17‐1 (Simon et al., 1986). We selected transconjugants of PD1222 by plating on LB agar plates supplemented with 50 μg mL^−1^ kanamycin and 100 μg mL^−1^ rifampicin. We confirmed the final plasmid constructions by PCR and DNA sequencing.

To construct the derivative strain of PD1222 that constitutively expresses tdTomato (red fluorescent protein), we first cloned the promoter region of the Pden_2763 gene (genomic positions 2,783,297–2,783,614), which is constitutively activated in *P. denitrificans* (Morinaga et al., 2018). Briefly, we PCR amplified the *tdTomato* gene from ptdTomato (Takara Bio, Kusatsu, Japan) with primers P2763_tdTomaF and tdTomaR and the upstream region of Pden_2763 with primers P2763F and P2763_tdTomaR (Table S3). We then joined the amplicons by overlapping PCR using primers P2763nestF and tdTomanestR (Table S3), digested plasmid pBBPML and the joined amplicons with *Hind*III and *Xba*I, and ligated the digested joined amplicons into the digested plasmid pBBPML using the HiFi DNA Assembly Master Mix (New England Biolabs, Ipswich, MA, USA). This resulted in the completed plasmid pBBPdentdtomato (Table S1). Finally, we replicated the completed plasmid in strain DH5α, delivered it into strain PD1222 by conjugation from strain S17‐1 (Simon et al., 1986), and selected for transconjugants of strain PD1222 by plating on LB agar plates amended with 50 μg mL^−1^ kanamycin and 100 μg mL^−1^ rifampicin. We confirmed the final plasmid construction by PCR and DNA sequencing.

### Quantification of oxygen, acetate, nitrogen oxides and nitrogen gas dynamics

We assessed the growth and substrate consumption/reduction capabilities of strain PD1222 when grown in oxic or anoxic liquid batch cultures using chemical and isotopic analytic methods. We first prepared minimal medium (MM) amended with 20 mM of Na^15^NO_3_ (Cambridge Isotope Laboratories, Tewksbury, MA, USA), where the MM consisted of 3.0 g L^−1^ Na_2_HPO_4_, 3.0 g L^−1^ (NH_4_)_2_SO_4_, 1.4 g L^−1^ KH_2_PO_4_, 0.3 g L^−1^ MgSO_4_7H_2_O, 0.3 g L^−1^ NaHCO_3_ and 50 mM acetate as the sole carbon and energy source. We further amended the MM with 3.5 mL L^−1^ of a trace element solution consisting of the following (g L^−1^): 9.0 of FeSO_4_7H_2_O, 9.0 of CaCl_2_, 1.8 of MnCl_2_2H_2_O, 1.5 of ZnSO_4_7H_2_O and 0.15 of MnCl_2_4H_2_O. We then decanted 10 mL aliquots of the MM into replicated 123 mL of glass serum vials, inoculated the vials with 100 μL of an overnight culture of strain PD1222, sealed the vials with butyl rubber stoppers and replaced the headspaces of all the vials with helium gas (purity >99.9%). For the experiments in oxic batch cultures, we then removed 35 mL of the helium gas and replaced the volume with 35 mL of oxygen gas (purity >99.9%). Finally, we incubated the vials at 30°C with shaking at 150 rpm for 48 h and measured optical densities at 600 nm (OD_600_) and the concentrations of oxygen, acetate, nitrate, nitrite, nitric oxide, nitrous oxide and nitrogen gas over time as described elsewhere (Oshiki et al., 2022). Briefly, we measured OD_600_ using an ARVO MX plate reader (Perkin‐Elmer, Waltham, MA, USA); oxygen using a GC‐8A gas chromatograph equipped with a thermal conductivity detector (Shimadzu, Kyōto, Japan); acetate, nitrate and nitrite using a CDD‐6A ion chromatograph (Shimadzu, Kyōto, Japan) equipped with an NI‐424 column (Shodex, Tokyo, Japan) and ^15^NO, ^15^N_2_O and ^15^N_2_ using a GCMS‐QP 2010 SE gas chromatograph‐mass spectrometer (Shimadzu, Kyōto, Japan).

### Flow cytometry

We quantified the per‐cell activities of the *nar*, *nir*, *nor* and *nos* promoters using flow cytometric analyses of the promoter‐reporter strains of PD1222 (Table S1). We first grew strain PD1222 carrying plasmid pRPOBE‐Pnar‐mNeonGreen, pRPOBE‐Pnir‐mNeonGreen, pRPOBE‐Pnor‐mNeonGreen or pRPOBE‐Pnos‐mNeonGreen (Table S1) in MM with and without 50 mM nitrate in oxic or anoxic liquid batch cultures at 30°C. After incubation for 10 h, such that the cells were in the exponential phase, we removed liquid aliquots, collected the cells by centrifugation, and washed and diluted the cells in carbon‐free phosphate‐buffered saline (PBS) solution. We then exposed the cells to ambient air for 1 h under non‐growing conditions (i.e., suspended in carbon‐free PBS) to induce the maturation of green fluorescent protein. Finally, we analysed the expression of each promoter (*nar*, *nir*, *nor* and *nos*) based on the intensity of green fluorescence using an SH800 flow cytometer (Sony Life Science Business, Yokohama, Japan). We excited green fluorescent protein with a 488‐nm laser and detected emission using a 525/50‐nm filter. We quantified green fluorescence for 100,000 cells from each sample and performed all data analyses with the SH800 software (Sony Life Science Business, Yokohama, Japan) and FlowJo software (V10.7) (FlowJo, Ashland, OR, USA).

### Colony growth experiments

We measured the surface‐associated growth of strain PD1222 by quantifying the radial extent of colony growth on agar plates. We first grew strain PD12222 overnight in oxic liquid TSB medium at 30°C and used this overnight culture to inoculate replicated LB agar plates, where we deposited 2‐μL drops onto the centres of separate LB agar plates (one drop per plate). We next incubated the LB agar plates at 30°C in an oxic or anoxic atmosphere and quantified the radial extent of colony growth using confocal laser scanning microscopy (CLSM) as described below. For the oxic atmosphere, we incubated the LB agar plates in ambient air. For the anoxic atmosphere, we transferred the LB agar plates into a glove box (Coy Laboratory Products, Grass Lake, MI, USA) filled with a nitrogen:hydrogen (97%:3%) gas mix.

We measured the extent of spatial intermixing of two subpopulations of strain PD1222 during colony growth using modified versions of protocols described in detail elsewhere (Goldschmidt et al., 2017). For our study, we first grew two overnight cultures of strain PD1222, where one carried plasmid pBBPdenzsGreen and the other carried plasmid pBBPdentdtomato (Table S1), in oxic liquid TSB medium at 30°C. We then adjusted the OD_600_ of the overnight cultures to 0.1, mixed them together at a ratio of 1:1 (pBBPdenzsGreen:pBBPdentdtomato), and deposited 2‐μL drops onto the centres of separate LB agar plates (one drop per plate). We finally incubated the LB agar plates at 30°C in an oxic or anoxic atmosphere as described above, exposed the LB agar plates to ambient air to induce maturation of the fluorescent proteins, and quantified the extent of spatial intermixing with CLSM as described below.

### Quantification of colony diameter and spatial intermixing

We used a TCS SP5 II confocal laser scanning microscope (Leica Microsystems, Wetzlar, Germany) equipped with a 2.5× HCX FL air immersion lens and a frame size of 512 × 512 (resulting in a pixel size of 0.0825 μm). We set the laser emission to 488 nm for the excitation of green fluorescent protein (encoded by the *zsGreen* gene) and to 514 nm for the excitation of red fluorescent protein (encoded by the *tdtomato* gene). We used the software package Fiji and Fiji plugins (https://fiji.sc) to quantify both the radial extent of colony growth and the spatial intermixing of different subpopulations from the CLSM images. To measure the radial extent of colony growth, we applied the Fiji default algorithm for image thresholding on a single fluorescence channel to binarize the images. We then quantified the diameter of the colony using the particle analysis feature in Fiji. For spatial intermixing, we used the method that we described in detail elsewhere (Ciccarese et al., 2022; Goldschmidt et al., 2017). Briefly, we measured the number of intersections between the background and information‐containing parts of the image using the Sholl plugin (Ferreira et al., 2014) of ImageJ (https://imagej.net). We applied a concentric windowing method starting at the initial deposition area and extending to the final colony periphery using a radial step size of 10 μm. For each radial increment, we calculated the intermixing index *I*_*r*_ as the number of intersections *N*_*r*_ divided by the expected number of intersections for a random spatial distribution of two populations at a given radius *r* as described in Equation (1) (Goldschmidt et al., 2017).

(1) $$I_{r}=\frac{N_{r}}{\mathrm{\pi r}/2}$$

### Computational modelling

We used CellModeller (Rudge et al., 2012; Rudge et al., 2013), which is a modular platform for individual‐based computational modelling, to investigate how interactions both between and within subpopulations affect the extent of spatial intermixing during colony growth. We simulated cell growth across a two‐dimensional plane (bacterial monolayer) that initially contained 100 cells of two types that only differed in their colour (labelled magenta or green). We homogenously deposited the cells across a circular space with random rotation in the *x*,*y* plane. The cells are rod‐shaped with a radius of 0.5 μm and a length of 1.5 μm to represent *P. denitrificans* and divide when reaching a length of 3.0 μm. We modelled the expansion of the colony using the integrator module that solves differential equations for intracellular chemical dynamics, as well as for a signalling module that can diffuse in and out of cells and around the extracellular space. We performed five independent simulations for each condition.

To model the growth rate during intermediate inhibition, we used a toxin (intermediate) signal‐dependent Monod‐type formulation for one or both cell types and their neighbours as described in Equations (2) and (3), respectively. We set the maximum specific growth rate (*μ*_max_) to 10 and varied the *K*_tox_ parameter between values of 0.005, 0.01, 0.05, 0.1 and 0.5 (Table S4). As the concentration of the toxin increases, it competes with the substrate, leading to a decrease in the growth rate. The parameter *i* determines the strength of substrate competition between a focal cell and its neighbours. Our execution of the model used the CLCrankNicIntegrator and GridDiffusion modules in Cellmodeller (Rudge et al., 2012).

(2) $$\mu =\mu_{\max}\left(1-\frac{iS_{A}}{K_{\mathrm{tox}}+iS_{A}}\right)$$

(3) $$\mu =\mu_{\max}\left(1-\frac{iS_{A}+iS_{B}}{K_{\mathrm{tox}}+iS_{A}+iS_{B}}\right)$$

We used Equations (4) and (5) to model the growth rate with and without intermediate inhibition, respectively, incorporating both toxin (intermediate) production and detoxification signals from each cell and its neighbours. The Monod‐type formulation includes the parameter *μ*_max_ and the variable *K*_tox_. To account for the detoxification ability, we added the parameter *K*_detox_ to the equations, which we set to 0.0001 for all simulations. As with the above models, the parameters used for these models are in Table S4 and our execution used the CLCrankNicIntegrator and GridDiffusion modules in Cellmodeller (Rudge et al., 2012).

(4) $$\mu =\mu_{\max}\left(1-\frac{iS_{A}}{K_{\mathrm{tox}}+iS_{A}}\frac{K_{\text{detox}}+iS_{A}}{K_{\text{detox}}}\right)$$

(5) $$\mu =\mu_{\max}\left(1-\frac{iS_{A}}{K_{\mathrm{tox}}+iS_{A}}\frac{K_{\text{detox}}+iS_{B}}{K_{\text{detox}}}\right)$$

### Quantitative and statistical analyses

We performed all statistical tests in the R software environment (R version 4.2.3) (https://www.r-project.org/). For each data set, we tested for homoscedasticity with the Bartlett test and normality with the Shapiro–Wilk test. We assessed statistical significance between means using two‐sample two‐sided Student's *t* tests. We used Student's *t* tests because our data sets generally do not significantly deviate from the assumptions of normality and homoscedasticity. We used Spearman rank‐correlation tests to test for pairwise associations between variables. We therefore did not make any assumptions regarding the linearity of the associations; rather, we tested more generally for monotonically increasing or decreasing associations. We used two‐sample two‐sided Kolmogorov–Smirnov tests to test whether the distributions of promoter activities differ between treatments. We reported the statistical test and the sample size (*n*) for each test in the results section, where all sample sizes are the number of independent biological replicates.

## RESULTS

### Accumulation of nitrogen oxide intermediates

**FIGURE 1:**
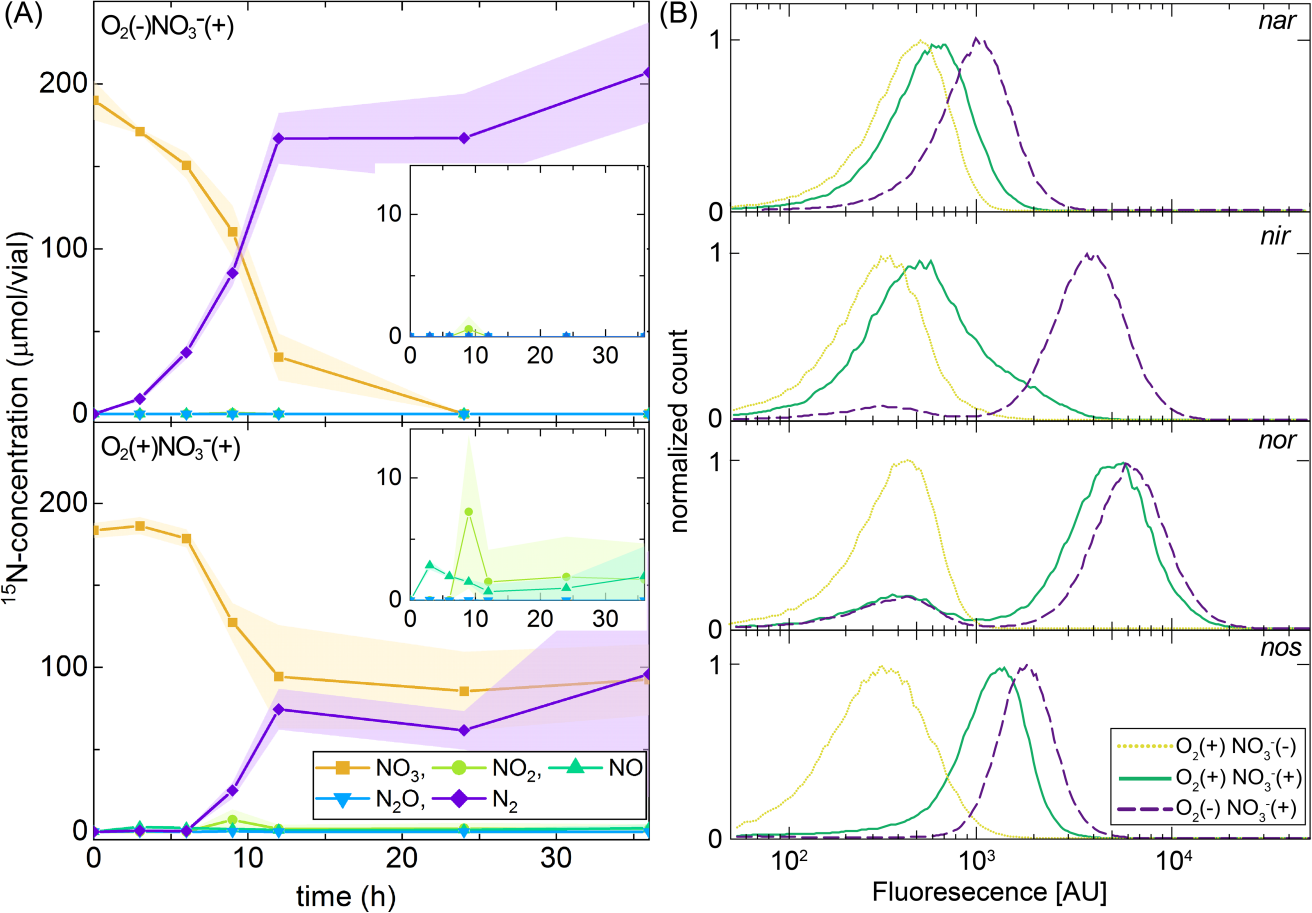
Dynamics of nitrogen oxide production and reduction by strain PD1222 in oxic and anoxic liquid batch cultures. (A) ^15^NO_3_^−^ reduction in anoxic (upper) and oxic (lower) liquid cultures at pH 7.5. Data points are averages of three independent biological replicates and the shaded regions are ± one standard deviation. Symbols and colours are for nitrate (square, yellow), nitrite (circle, yellow‐green), nitric oxide (upper triangle, green), nitrous oxide (lower triangle, blue) and nitrogen gas (diamond, purple). For incubations in oxic liquid cultures, the oxygen concentrations in the headspace were ~10% (v/v) throughout the duration of the experiment as determined by gas chromatography (Figure S1). (B) Single‐cell analysis of the activities of the *nar*, *nir*, *nor* and *nos* promoters quantified by flow cytometry. Each promoter‐reporter strain was incubated individually and the promoter activity levels were measured for 100,000 cells. The normalized count is the number of events at each fluorescence intensity normalized by the maximum number of events. Lines and colours are for O_2_(+) NO_3_^−^(−) (dotted line, yellow), O_2_(+) NO_3_^−^(+) (solid line, green) and O_2_(−) NO_3_^−^(+) (dashed line, purple). Data were taken after 10 h of incubation at 30°C when cells were in the exponential growth phase.

We first tested whether the accumulation patterns of nitrogen oxide intermediates during denitrification by strain PD1222 are different in oxic and anoxic environments. To test this, we tracked the production and reduction dynamics of nitrogen oxides during growth in liquid cultures at pH 7.5 using isotopic nitrogen measurements. We observed the complete reduction of nitrate to nitrogen gas in both oxic and anoxic liquid cultures (Figures 1A and S1), indicating that all components of the denitrification pathway were synthesized and functionally active in both environments. However, nitrite reduction did not begin until the gas‐phase oxygen concentration was reduced to approximately 10% (Figure S1A–H), and we therefore refer to this as denitrification in low oxic environments. We further found that the maximum rates of nitrate reduction were statistically identical in low oxic and anoxic liquid cultures (two‐sample two‐sided *t* test; *p* = 0.15, *n* = 3) (Figures 1A and S1C), indicating that the nitrate reductase had approximately equivalent activity levels/abundances in the two environments. We also observed a significantly lower total extent of nitrate reduction in low oxic liquid cultures when compared to anoxic liquid cultures at the end of the experiment (two‐sample two‐sided *t* test; *p* = 0.018, *n* = 3) (Figure 1A). This was due to carbon limitation; the carbon was partitioned between denitrification and oxygen respiration in the low oxic liquid cultures but was exclusively directed to denitrification in the anoxic liquid cultures (Figure S1B), thus resulting in a lower total extent of nitrate reduction in the low oxic environment (Figure S1C). Importantly, we also observed significantly higher maximum concentrations of nitrite (two‐sample two‐sided *t* test; *p* = 0.029, *n* = 3) and nitric oxide (two‐sample two‐sided *t* test; *p* = 0.005, *n* = 3) in the low oxic liquid cultures when compared to the anoxic liquid cultures (Figures 1A and S1D,E). Thus, nitrogen oxide intermediates accumulate to higher concentrations in the low oxic environment. Because the maximum rates of nitrate reduction were statistically indistinguishable in the low oxic and anoxic liquid cultures (Figure 1A), we attribute the increased accumulation of nitrogen oxide intermediates in the low oxic liquid cultures to reductions in the activity levels/abundances of downstream enzymes.

### Expression of nitrogen oxide reductase‐encoding genes

To further test the hypothesis that the increased accumulation of nitrogen oxide intermediates in the low oxic liquid cultures at pH 7.5 was due to reductions in the activity levels/abundances of downstream enzymes, we quantified the activity levels of the *nar*, *nir*, *nor* and *nos* promoters, which encode for the four nitrogen oxide reductases involved in the complete denitrification pathway. The per‐cell activity levels of these reductases can be heterogeneous in *P. denitrificans* populations, where a single clonal population can consist of subpopulations with different expression levels (Lycus et al., 2018). We therefore quantified the activity levels of the *nar*, *nir*, *nor* and *nos* promoters at the single‐cell level by constructing promoter‐reporter strains, where we inserted each promoter into a separate plasmid at a location immediately upstream of a green fluorescent protein‐encoding gene. We then quantified the activity level of each promoter in low oxic and anoxic liquid cultures by measuring the intensity of green fluorescence via flow cytometry.

We observed significant differences in the promoter activity levels in the low oxic and anoxic liquid cultures (Figure 1B). We observed statistically significant but quantitatively modest differences in the activity levels of the *nar*, *nor* and *nos* promoters in the low oxic and anoxic liquid cultures (two‐sample two‐sided Kolmogorov–Smirnov tests; *D* < 0.5, *p* = 0), where the activity levels of all three promoters were somewhat repressed by 27%–40% in the low oxic environment (Figure 1B). In contrast, we observed a statistically significant and large difference in the activity level of the *nir* promoter in the low oxic and anoxic liquid cultures (two‐sample two‐sided Kolmogorov–Smirnov test; *D* = 0.8, *p* = 0), where the activity level of the *nir* promoter was repressed by nearly an order of magnitude (85%) in the low oxic environment (Figure 1B). Thus, the relative activity level of the *nar* to *nir* promoters is higher in the low oxic environment than in the anoxic environment. This would result in a larger relative abundance of the nitrate to nitrite reductases and a consequent increase in the accumulation of nitrite in the low oxic environment, which is what we observed experimentally (Figure 1A). We therefore conclude that the increased accumulation of nitrogen oxide intermediates in low oxic liquid cultures is indeed caused by reductions in the activity levels/abundances of downstream enzymes, and in particular of nitrite reductase. Stated alternatively, low oxic conditions are sufficient to induce robust activity of the *nar* promoter but only minimal activity of the *nir*, *nor* and nos promoters.

### Growth is inhibited by denitrification in oxic environments at low pH

The accumulation of nitrite and nitric oxide can have a severe environment‐dependent inhibitory effect on microbial growth (Sijbesma et al., 1996; Zhou et al., 2011; Wilbert & Newman, 2022), particularly in low‐pH environments that can exacerbate the toxicity pathways of both nitrite and nitric oxide (Hartop et al., 2017; Pacher et al., 2007; Zhou et al., 2011). We therefore hypothesized that growth of strain PD1222 in low oxic environments should be repressed at low pH because of increased nitrite and nitric oxide accumulation, while growth in anoxic environments should be unaffected by pH because of the absence of observable nitrite and nitric oxide accumulation (Figure 1A). To test this hypothesis, we quantified growth in low oxic and anoxic liquid cultures at different pH conditions. Based on previous studies (Lilja & Johnson, 2016), we selected pH 6.5 to induce the growth‐inhibiting effects of nitrite and nitric oxide and pH 7.5 to repress those effects.

**FIGURE 2:**
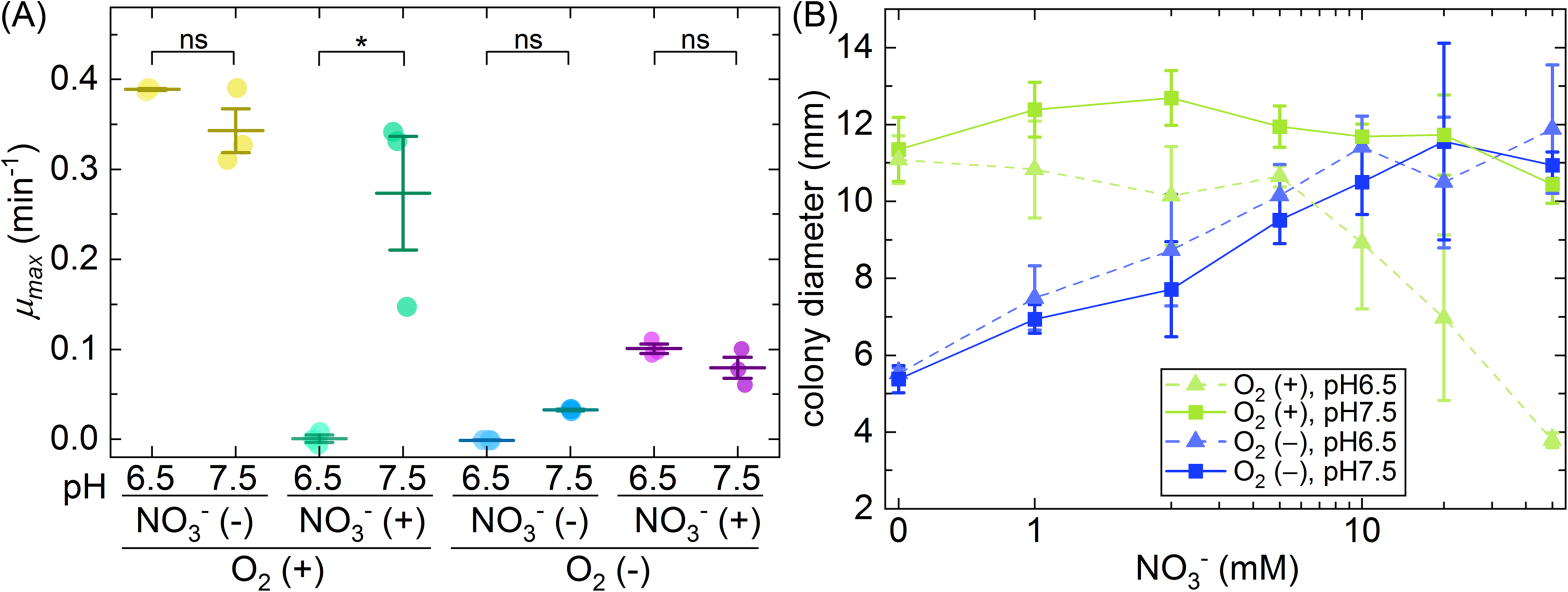
Effect of pH on growth in oxic and anoxic environments. (A) The maximum specific growth rate of strain PD1222 (*μ*_max_) in oxic or anoxic liquid batch cultures amended with or without nitrate. The *μ*_max_ was calculated from five consecutive data points coinciding with the most rapid period of growth for each individual culture. Each data point is the measurement for an independent experimental replicate (*n* = 3). The middle horizontal line is the mean and the upper and lower horizontal lines are ± one standard deviation. Asterisks indicate statistically significant differences in the means of the labelled groups calculated with two‐sample two‐sided *t* tests (ns, no significant difference; *, *p* < 0.05). (B) Colony growth as a function of the nitrate concentration. Colonies were grown on LB agar plates (one colony per plate) at pH 6.5 or 7.5 and incubated in an oxic or anoxic atmosphere for 5 days at 30°C. Data points are the means and error bars are ± one standard deviation from three independent biological replicates. LB, lysogeny broth.

We found that the accumulation of nitrite and nitric oxide during denitrification in low oxic liquid cultures at low pH does indeed repress growth. In low oxic liquid cultures in the absence of nitrate, we did not observe any significant effect of the pH on the maximum specific growth rate (two‐sample two‐sided *t* test; *p* = 0.198, *n* = 3) (Figure 2A), which is expected because there is no production of nitrite or nitric oxide in the absence of nitrate. This provides evidence that pH itself does not have any effect on growth over the experimentally manipulated pH range (6.5–7.5). In anoxic liquid cultures in the presence of nitrate, we also did not observe any significant effect of the pH on the maximum specific growth rate (two‐sample two‐sided *t*‐test; *p* = 0.205, *n* = 3) (Figure 2A), which is again expected because nitrite and nitric oxide are produced but do not accumulate in this environment (Figure 1A). In the presence of nitrate in low oxic liquid cultures, however, we observed a significant reduction in the maximum specific growth rate at pH 6.5 (two‐sample two‐sided *t*‐test; *p* = 0.049, *n* = 3) (Figure 2A). In this environment, nitrite and nitric oxide are produced, accumulate, and have increased growth‐inhibiting effects (Figure 1A). Thus, the increased accumulation of nitrite and nitric oxide during denitrification in a low oxic environment does indeed have a significant effect on growth at low pH.

We next tested whether the negative effect of denitrification on growth in oxic environments is specific to cells growing in liquid culture or whether it also occurs for cells growing associated with surfaces. To test this, we measured surface‐associated growth in an anoxic or oxic atmosphere as the radial extent of colony expansion on agar plates for different pH conditions. For agar plates incubated in an anoxic atmosphere, the colony diameter at the end of the experiment depended on the exogenously supplied concentration of nitrate (two‐sided Spearman test; *r* = 0.964, *p* = 0.003, *n* = 7) but was unaffected by the pH (two‐sample two‐sided *t* test at 50 mM NO_3_^−^; *p* = 0.576, *n* = 3) (Figure 2B). This is expected because nitrite and nitric oxide are produced but do not accumulate in anoxic environments (Figure 1A). For agar plates incubated in an oxic atmosphere at pH 7.5, the colony diameter at the end of the experiment was independent of the concentration of nitrate (two‐sided Spearman test; r = −0.393, *p* = 0.396, *n* = 7) (Figure 2B). This demonstrates that denitrification by colonies in an oxic atmosphere at pH 7.5 had a negligible effect on growth, which is expected as there was a continuous supply of oxygen from the atmosphere and nitrite and nitric oxide do not have growth‐inhibiting effects at this pH (Figure 2A). In agar plates incubated in an oxic atmosphere at pH 6.5, however, the colony diameter at the end of the experiment was significantly reduced as the concentration of nitrate increased (two‐sided Spearman test; *r* = −0.964, *p* = 0.003, *n* = 7) (Figure 2B). This demonstrates that denitrification by colonies in an oxic atmosphere at pH 6.5 had a significant negative effect on growth, which is expected because nitrite and nitric oxide are produced, accumulate (Figure 1A), and have significant growth‐inhibiting effects at this pH (Figure 2A). Thus, as we observed in liquid culture, the increased accumulation of nitrite and nitric oxide during denitrification in oxic environments also has a significant negative effect on surface‐associated growth at low pH.

### pH determines evolutionary potential

We finally tested whether the accumulation of nitrite and nitric oxide during colony growth in an oxic atmosphere could, in principle, modulate the evolutionary potential of strain PD1222. Briefly, as a population of cells expands across a surface during colony growth, only a few individuals lying close to the colony periphery will contribute to further growth (Goldschmidt et al., 2017; Hallatschek et al., 2007; Hallatschek & Nelson, 2010; Mitri et al., 2015). If more of these individuals contribute to colony growth, then more of the standing genetic diversity within the initial population will be preserved. This results in more genetic targets for natural selection to act upon and a higher evolutionary potential. We can experimentally estimate the amount of the initial standing genetic diversity that is preserved during colony growth by growing colonies consisting of mixtures of two subpopulations that are genetically and phenotypically identical except that they express different fluorescent proteins (Goldschmidt et al., 2017; Hallatschek et al., 2007; Hallatschek & Nelson, 2010). The two subpopulations will segregate into sectors during colony growth, where each sector derives from a few individuals from the initial population (Goldschmidt et al., 2017; Hallatschek et al., 2007; Hallatschek & Nelson, 2010). Thus, the number of sectors that persist during colony growth is a proxy measure for how much of the initial standing genetic diversity is preserved.

**FIGURE 3:**
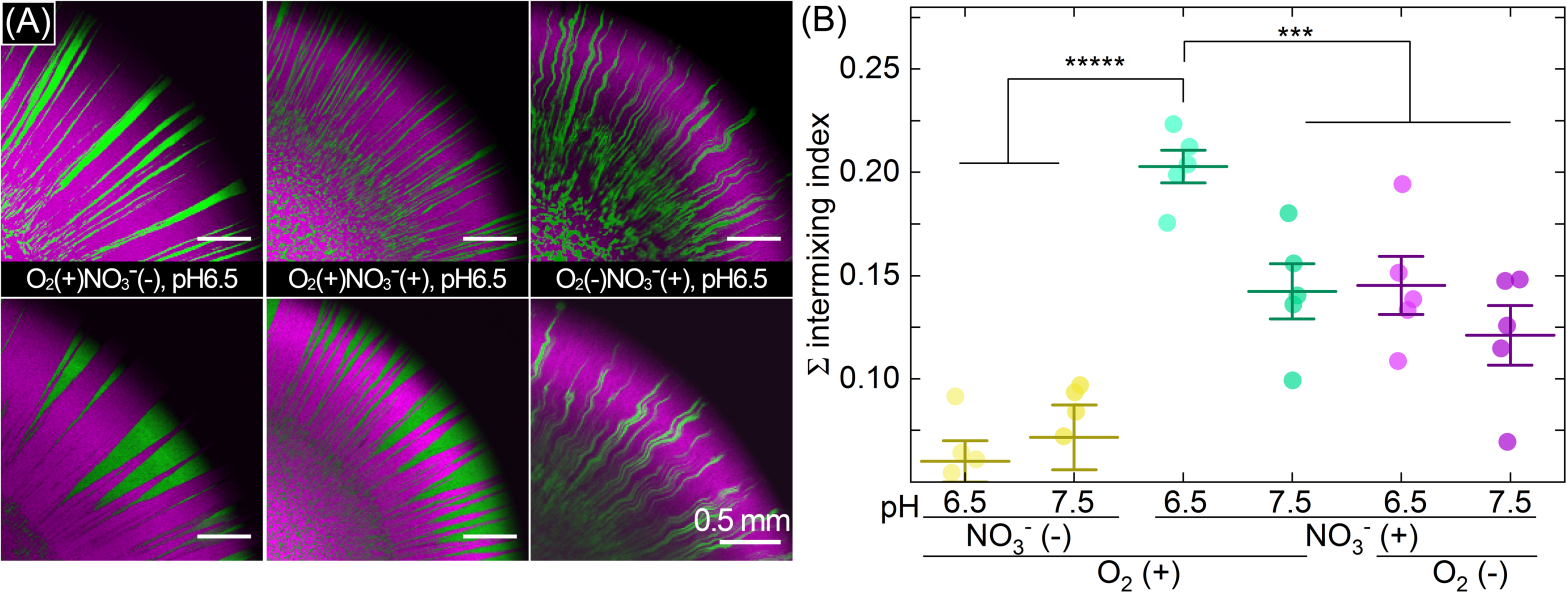
Effect of pH on spatial intermixing during colony growth in an oxic or anoxic atmosphere. (A) Representative CLSM images of quartiles from colonies growing on LB agar plates amended with or without 50 mM nitrate in an oxic or anoxic atmosphere. The colonies consist of two subpopulations of strain PD1222, where one expresses red (appears magenta here) while the other expresses green fluorescent protein. The images were taken after 5 days of incubation at 30°C. (B) The intermixing index of the two subpopulations at the final colony periphery (50 μm circular band positioned at the colony periphery). Each data point is the measurement for an independent experimental replicate (*n* = 5). The middle horizontal line is the mean and the upper and lower horizontal lines are ± one standard deviation. Asterisks indicate statistically significant differences in the means of the labelled groups calculated with two‐sample two‐sided *t* tests (***, *p* < 0.001; *****, *p* < 0.00001). CLSM, confocal laser scanning microscopy; LB, lysogeny broth.

To test whether the accumulation of nitrite and nitric oxide in an oxic atmosphere can modulate evolutionary potentials, we mixed two subpopulations of strain PD1222 that express either red or green fluorescent protein but are otherwise genetically and phenotypically identical and grew them on agar plates. As expected and consistent with previous studies (Goldschmidt et al., 2017; Hallatschek et al., 2007; Hallatschek & Nelson, 2010), the two subpopulations segregated into sectors during colony growth (Figure 3A). We then measured the intermixing index of the spatial patterns by quantifying the circumference‐normalized number of sectors that emerge along the colony periphery (Figure S2). For colonies grown in an anoxic atmosphere in the presence of nitrate, we did not observe any significant effect of the pH on the intermixing index (two‐sample two‐sided *t* test; *p* = 0.208, *n* = 5) (Figure 3B). For colonies grown in an oxic atmosphere in the presence of nitrate, in contrast, we observed a significant effect of the pH on the intermixing index, where the intermixing index was higher at low pH (two‐sample two‐sided *t* test; *p* = 0.006, *n* = 5) (Figure 3B). We therefore conclude that denitrification in an oxic environment at low pH results in higher spatial intermixing, which corresponds to more individuals from the initial standing population contributing to colony growth, higher levels of genetic diversity for selection to act upon, and higher evolutionary potentials.

### Inter‐ and intra‐specific interactions control spatial intermixing

**FIGURE 4:**
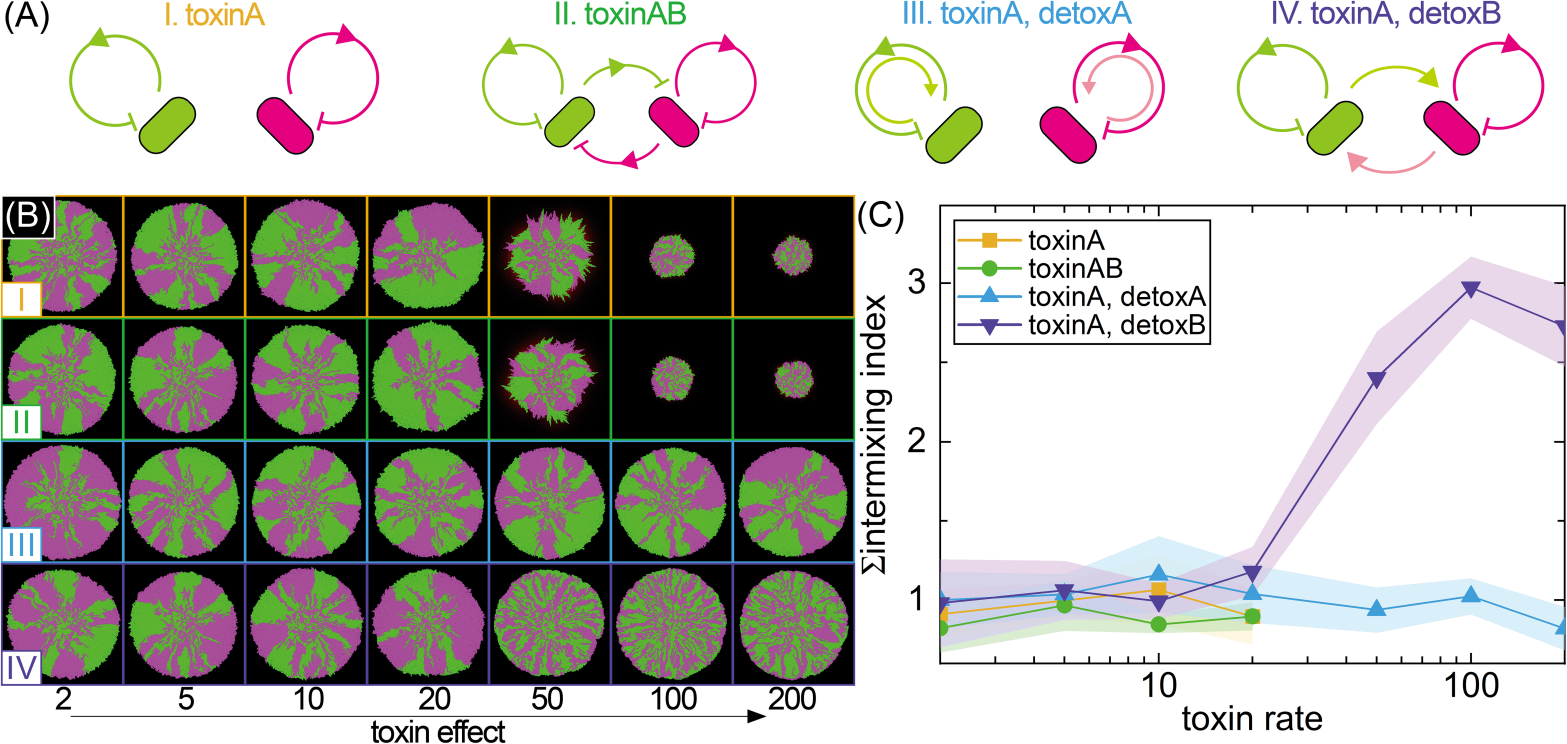
Effect of interactions between and within subpopulations and the magnitude of intermediate toxicity on spatial intermixing during colony growth. (A) The four models that we investigated in this study. We simulated growth inhibition by producing and receiving signals (intermediates) within cells of the same colour (I), as well as cells with different colours (II). Additionally, we incorporate a detoxification factor by producing and receiving signals within cells of the same colour (III) and cells with different colours (IV), both based on the inhibition process itself. (B) Representative simulations for the four different models as a function of the strength of the toxic effect of the intermediate. (C) The intermixing index at the final colony periphery for the simulation results. For (B) and (C), the toxic effects were set by 1/*K*_tox_ and the detox factor (consumption of the intermediate) was consistent across all simulations (*K*_detox_ = 0.001). Data points are the averages from five independent simulations and the shaded regions are ± one standard deviation.

Why is there more spatial intermixing (i.e., more sectors along the colony periphery) during colony growth in an oxic atmosphere at low pH when nitrite and nitric oxide accumulate and have a strong growth‐inhibiting effect? To address this question and identify a plausible underlying mechanism, we used an individual‐based computational framework to simulate colony growth. The framework allows us to modify the growth‐inhibiting effect of an intermediate (simulates nitrite and/or nitric oxide) and quantify the consequences on spatial intermixing and sector formation. We tested four different models for how subpopulations could interact with each other and with themselves (Figure 4A; designated as models I–IV). Model I assumes that each subpopulation inhibits itself via the production of the intermediate but does not inhibit the other subpopulation. This would occur if the spatial rage to which the intermediate has a growth‐inhibiting effect were short and on the order of a few cell lengths (i.e., the intermediate produced by a cell would only affect that cell and perhaps a few immediately neighbouring cells, which are likely to be kin). Model II assumes that each subpopulation inhibits both itself and the other subpopulation via the production of the intermediate. This would occur if the spatial range to which the intermediate has a growth‐inhibiting effect were longer than a few cell lengths (i.e., the intermediate produced by a cell would not only affect immediately neighbouring kin cells but also more distantly located non‐kin cells). Model III is identical to model I except that immediately neighbouring kin cells can consume the intermediate and alleviate its growth‐inhibiting effect. Finally, model IV is also identical to model I except that more distantly located non‐kin cells can consume the intermediate and alleviate its growth‐inhibiting effect.

Our simulations revealed two important outcomes. First, when the intermediate cannot be consumed (models 1 and 2), increasing its growth‐inhibiting effect causes colony growth to slow down and eventually stop (Figure 4B). In contrast, when the intermediate can be consumed (models 3 and 4), colony growth can persist, albeit more slowly than when the intermediate does not have a growth‐inhibiting effect (Figure 4B). Second, regarding the effect of intermediate toxicity on spatial intermixing, we found that only one of the four tested models generates results that are qualitatively consistent with our experimental observations. For models 1–3, we did not observe a relationship between the amount of spatial intermixing that emerges during colony growth and the magnitude of the growth‐inhibiting effect of the intermediate (two‐sided Spearman tests; *r* = −0.46 to −0.40, *p* > 0.30) (Figure 4C), which is counter to our experimental observations regarding the effect of pH (and thus nitrite and nitric oxide toxicity) on spatial intermixing (Figure 3). For model 4, in contrast, we observed a positive relationship between the amount of spatial intermixing that emerges during colony growth and the magnitude of the growth‐inhibiting effect of the intermediate (two‐sided Spearman test; *r* = 0.929, *p* = 0.007) (Figure 4C), which is qualitatively consistent with our experimental observations regarding the effect of pH on spatial intermixing (Figure 3). Thus, we conclude that local growth inhibition by the production of an intermediate by neighbouring kin cells combined with the alleviation of growth inhibition by the consumption of the intermediate by more distantly located non‐kin cells can cause spatial intermixing to increase with the growth‐inhibiting effect of the intermediate.

## DISCUSSION

Our findings shed light on the ecological and evolutionary significance of the accumulation of nitrogen oxide intermediates by denitrifying microorganisms in oxic environments. While continuous cultures maintained at controlled dissolved oxygen concentrations can inhibit nitrate reduction and denitrification gene regulation (Giannopoulos et al., 2017), we demonstrate significant nitrate reduction and denitrification activity even in the presence of low concentrations of oxygen. We suspect that the denitrification activity by *P. denitrificans* is triggered by low oxygen concentrations, as the peak observed denitrification activity and the maximal nitrite and nitric oxide accumulation concentrations occur when the gas‐phase oxygen concentration declines to 10%. Furthermore, we found that denitrification in low oxic environments results in greater accumulation of nitrite and nitric oxide than in anoxic environments (Figure 1A), with severe negative consequences on the growth of denitrifying populations in low pH conditions, when nitrite and nitric oxide have increased growth‐inhibiting effects (Figure 2). These findings are consistent with a previous study that also reported the pH‐dependent inhibition of *P. denitrificans* by nitrite in oxic environments (Hartop et al., 2017); however, that study did not investigate the accumulation of intermediates during denitrification in oxic environments.

We demonstrated that the increased accumulation of nitrite and nitric oxide is caused by changes in the expression/activity levels of downstream reactions within the denitrification pathway (Figure 1B). The denitrification process of *P. denitirificans* is regulated by the FNR‐type regulator proteins FnrP, NarR and NNR, where FnrP responds to anoxic conditions, N_2_O and NO (Hassan, Bergaust, et al., 2016; Hassan, Qu, et al., 2016; Lee et al., 2006). In particular, the reduction of nitrate to nitrite is especially relevant, as existing studies indicate that nitrate reduction, mediated by Nar, is inhibited by oxygen (Zumft, 1997). This inhibition is likely due to the suppression of nitrate transport by NarK and the reduced promoter activity of *narK* (Alefounder & Ferguson, 1980; Wood et al., 2001). In our liquid batch experiments, we speculate that FnrP was active while NNR was not under low oxygen conditions, suggesting that FnrP activation of the *nar* promoter does not require strict anoxic conditions. Furthermore, another nitrate reductase, Nap, can additionally become relevant when considering nitrate reduction in the presence of oxygen (Bell et al., 1990; Ji et al., 2015; Sears et al., 1993). However, in our experiments, the key quantity is the relative activity of the *nar* to the *nir* promoter, which is significantly greater in low oxic than in anoxic environments (Figure 1B). This creates a bottleneck in low oxic environments, where there is insufficient nitrite reductase to keep up with the pace of nitrite production by nitrate reductase (Figure 1B). If Nap were also induced in low oxic environments, this would exacerbate the bottle neck and cause even more nitrite and nitric oxide to accumulate. We do note that because control of the relative activity of the *nar* to *nir* promoters appears to occur at the transcriptional level (Figure 1B), the accumulation of nitrite and nitric oxide in low oxic environments may be an evolvable trait, where prolonged propagation at low pH could select for new genotypes with more balanced expression/activity levels of the *nar* and *nir* promoters. Future experimental evolution studies would be useful to test this expectation (McDonald, 2019).

We further found that the accumulation of nitrite and nitric oxide in oxic environments increases the number of individuals from the initial population that contributes to colony growth and expansion (Figure 3). As more individuals contribute to colony growth, more of the initial standing genetic diversity is likely to be preserved, which would increase the number of genetic targets for selection to act upon and increase the evolutionary potential of the population. Our findings are consistent with a previous study of the bacterium *Pseudomonas stutzeri* (Goldschmidt et al., 2018). In that study, the authors conducted experiments with a nitrite cross‐feeding consortium, where one strain reduces nitrate to nitrite (the producer) while the other reduces nitrite (the consumer) (Goldschmidt et al., 2018). They found that more individuals of the producer contribute to colony growth and expansion as the pH decreases and the toxic effects of nitrite increase (Goldschmidt et al., 2018), which is consistent with our observations (Figure 3). The authors argued that these effects are caused by differences in effective population sizes (i.e., the number of individuals that are actively growing) (Goldschmidt et al., 2018). Briefly, increased nitrite and nitric oxide toxicity will slow the consumption of nitrate, which will allow nitrate to diffuse and penetrate further into the colony. This increases the effective population size, which in turn dampens the effects of ecological drift (Goldschmidt et al., 2018). These arguments are entirely applicable to our experiments also, as the primary substrate nitrate diffuses into the colony from the periphery. Thus, the idea that intermediate toxicity can determine the number of individuals that contribute to colony growth and expansion, and in turn modulate evolutionary potentials, appears to be generalizable to different species and interaction types. Nitrogen oxide toxicity may therefore be an important factor affecting the evolutionary processes acting on and the life histories of denitrifying microorganisms.

We further used individual‐based modelling to identify plausible ecological mechanisms for why intermediate toxicity affects the number of individuals that contribute to colony growth and expansion. We formulated and tested four models that make different assumptions regarding how subpopulations interact with each other (Figure 4A), yet only one of them (model 4) produces results that are qualitatively consistent with our experiments (Figure 4C). Why is this? Model 4 assumes that intermediate toxicity predominantly occurs within subpopulations over short distances while intermediate consumption/detoxification can occur between subpopulations over longer distances. We believe that these assumptions best mimic our experimental system for several reasons. First, models 1 and 2 do not consider intermediate consumption/detoxification (Figure 4A), and therefore do not capture the processes of nitrite and nitric oxide reduction by microorganisms that are capable of the complete denitrification process. Thus, their poor performance is not unexpected. Models 3 and 4 both assume that the toxic intermediates produced by a focal cell will disproportionately affect that particular focal cell (Figure 4A). This is consistent with basic mass transfer considerations. Nitrite and nitric oxide concentrations will be highest immediately adjacent to the cells that produce those intermediates due to a lack of complete mixing in spatially structured systems. This is also true in completely mixed systems due to the laminar boundary layers surrounding individual cells (Lilja & Johnson, 2016). Thus, the toxic effects caused by the intermediates produced by each individual cell will disproportionately affect that particular cell (Lilja & Johnson, 2016). However, because nitrite and nitric oxide accumulate locally in oxic environments (Figure 1A), they have the opportunity to diffuse away from the producing cell and be reduced by more distantly located cells, which are less likely to be kin. This is in essence what model 4 simulates; the toxic effects of intermediates predominantly act locally on the producing cell, while intermediate consumption/detoxification can occur by more distantly located cells that are less likely to be kin (Figure 4A). Thus, model 4 best considers how mass transfer aspects are likely to control the types of interactions that occur both within and between subpopulations.

Our study may have implications for understanding the maintenance and promotion of diversity within microbial communities. A previous study demonstrated that the transient accumulation of nitrogen oxide intermediates during growth of a completely denitrifying population creates a niche for a metabolically specialized population to invade into and occupy, thus promoting and maintaining diversity (Dolinšek et al., 2022). In our case, we observe the transient accumulation of nitrite and nitric oxide, and such transient accumulation could therefore create a niche for a nitrite‐ and/or a nitric oxide‐reducing metabolic specialist (note that nitric oxide, while being a redox‐active metabolite and a cellular toxin, also has a role in energy conservation in *Pseudomonas aeruginosa* [Wilbert & Newman, 2022], and the accumulation of nitric oxide may therefore represent a growth‐supporting niche). Our data, therefore, would lead to the prediction that denitrification in oxic environments is more likely to promote the maintenance of collections of metabolically specialized denitrifying microorganisms (enabled by the accumulation of nitrogen oxide intermediates), and thus promote diversity, than complete denitrification in anoxic environments.

Our study may also have practical implications for various applications of denitrifying microorganisms in low oxic environments, including in bioremediation, wastewater treatment, and agriculture. *P. denitrificans* is an effective denitrifier even in low oxic environments, yet it will accumulate nitrite and nitric oxide if certain sets of environmental conditions are met. In processes operating at high pH, the accumulation of nitrogen oxides may be of no consequence to system performance. However, if the pH is reduced below 7 when nitrite and nitric oxide can become severely toxic, then one needs further information regarding the accumulation of nitrite and nitric oxide in that particular system, and information regarding how one could reduce intermediate accumulation by modifying the pH or other environmental conditions.

## CONCLUSIONS

Our study provides insights into the ecological and evolutionary implications of denitrification in low oxic environments. We highlight the higher accumulation of nitrogen oxide intermediates, their inhibitory effect on microbial growth in low‐pH environments, and their potential to influence the genetic composition and evolutionary potential of denitrifying populations. Understanding the factors influencing nitrogen oxide accumulation and their effects on microbial communities is therefore important for the effective prediction, management and optimization of the nitrogen cycle in both engineered and natural systems.

## AUTHOR CONTRIBUTIONS

**Kohei Takahashi:** Conceptualization (equal); data curation (equal); funding acquisition (equal); investigation (lead); methodology (equal); writing – original draft (equal); writing – review and editing (equal). **Mamoru Oshiki:** Funding acquisition (supporting); investigation (supporting); methodology (supporting); writing – review and editing (supporting). **Chujin Ruan:** Data curation (equal); software (lead); writing – review and editing (supporting). **Kana Morinaga:** Investigation (supporting); writing – review and editing (supporting). **Masanori Toyofuku:** Methodology (supporting); writing – review and editing (supporting). **Nobuhiko Nomura:** Conceptualization (supporting); funding acquisition (equal); methodology (supporting); writing – review and editing (supporting). **David Johnson:** Conceptualization (equal); funding acquisition (equal); project administration (lead); resources (equal); supervision (lead); writing – original draft (equal); writing – review and editing (equal).

## CONFLICT OF INTEREST STATEMENT

The authors declare no conflict of interest.

## Supporting information


**DATA S1.** Supporting Information.

## Data Availability

All data and code generated in this study are publicly available in the Eawag Research Data Institutional Collection (ERIC) repository (https://opendata.eawag.ch/) at the following doi: https://doi.org/10.25678/0009B3.
