# Supplementary material for: Denitrification in low oxic environments increases the accumulation of nitrogen oxide intermediates and modulates the evolutionary potential of microbial populations
Source: Environ Microbiol Rep. 2023 Nov 30;16(1):e13221. doi: 10.1111/1758-2229.13221 (PMC10866065; doi:10.1111/1758-2229.13221)
Supplement: Supplementary file 1 — DATA S1. Supporting Information. [file EMI4-16-e13221-s001.docx]

# **SUPPORTING INFORMATION**

**Denitrification in low oxic environments increases the accumulation of nitrogen oxide intermediates and modulates the evolutionary potential of microbial populations**

Kohei Takahashi^1,2*^, Mamoru Oshiki^3^, Chujin Ruan^2^, Morinaga Kana^4^, Masanori Toyofuku^5,6^, Nobuhiko Nomura^5,6^, and David R. Johnson^2,7*^

^1^Graduate School of Sciences and Technologies, University of Tsukuba, Tsukuba, Ibaraki 305-8572, Japan; ^2^Department of Environmental Microbiology, Swiss Federal Institute of Aquatic Science and Technology (Eawag), 8600 Dübendorf, Switzerland; ^3^Division of Environmental Engineering, Faculty of Engineering, Hokkaido University, Sapporo, Hokkaido 060–8628, Japan; ^4^Bioproduction Research Institute, National Institute of Advanced and Industrial Science and Technology (AIST), Tsukuba, Ibaraki 305-8566, Japan; ^5^Faculty of Life and Environmental Sciences, University of Tsukuba, Tsukuba, Ibaraki 305-8577, Japan; ^6^Microbiology Research Center for Sustainability, University of Tsukuba, Tsukuba, Ibaraki 305–8572, Japan; ^7^Institute of Ecology and Evolution, University of Bern, 3012 Bern, Switzerland.

**Correspondence:**

Kohei Takahashi; takahashi.kohei.sj@alumni.tsukuba.ac.jp

David R. Johnson; david.johnson@eawag.ch

**This file includes:**

Tables S1-S4

Figures S1 and S2

Supporting References

# **Table S1**

# Strains and plasmids used in this study.

##
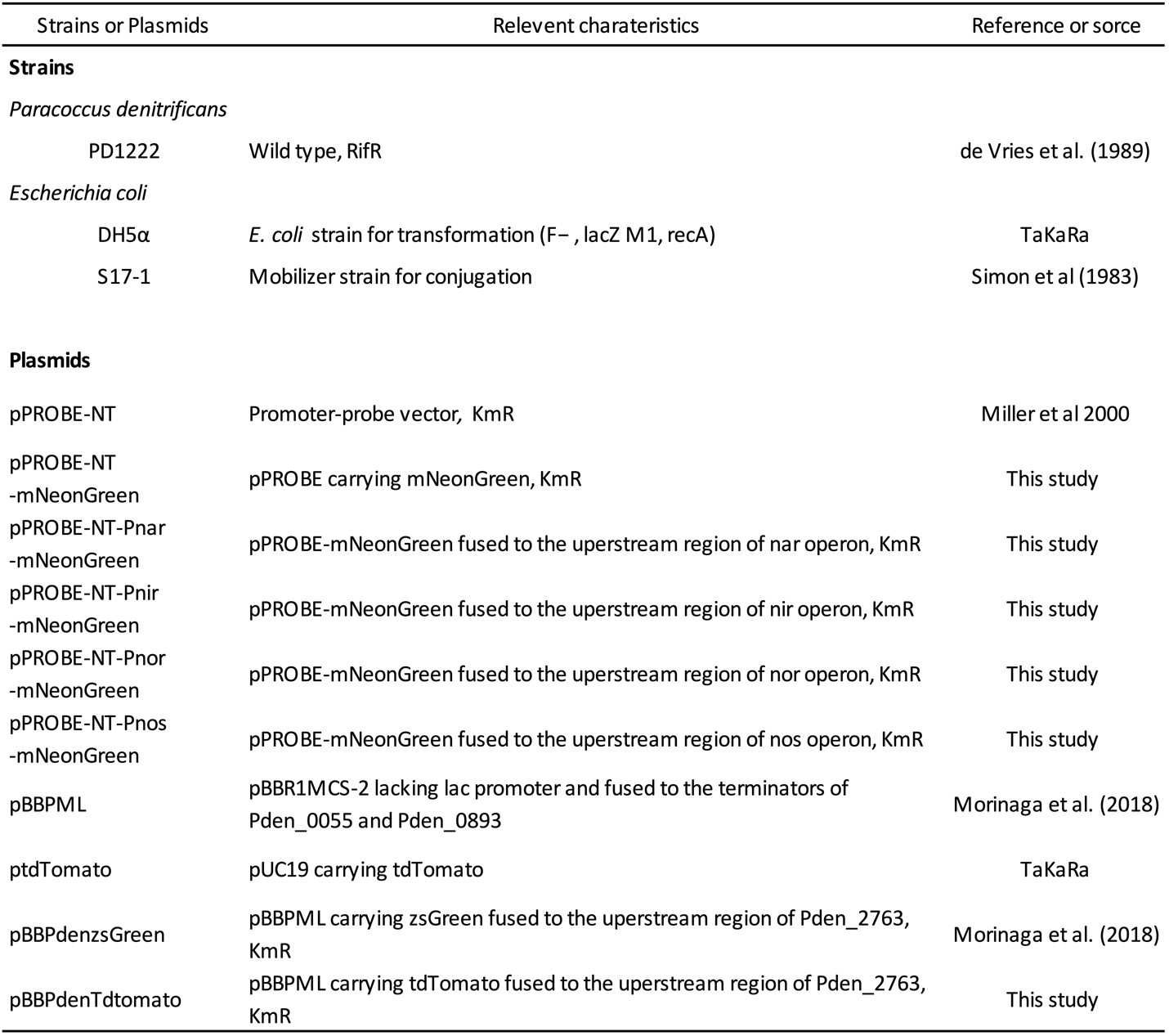


## **Table S2**

## Sequence of the *mNeonGreen* gene synthesized in this study.

## **Table S3**

## Primer sequences used in this study. The underlined sequences are those used for Infusion cloning.

**Table S4**

Model parameters used for individual-based computational simulations. Hyphens indicate arbitrary units.

## **
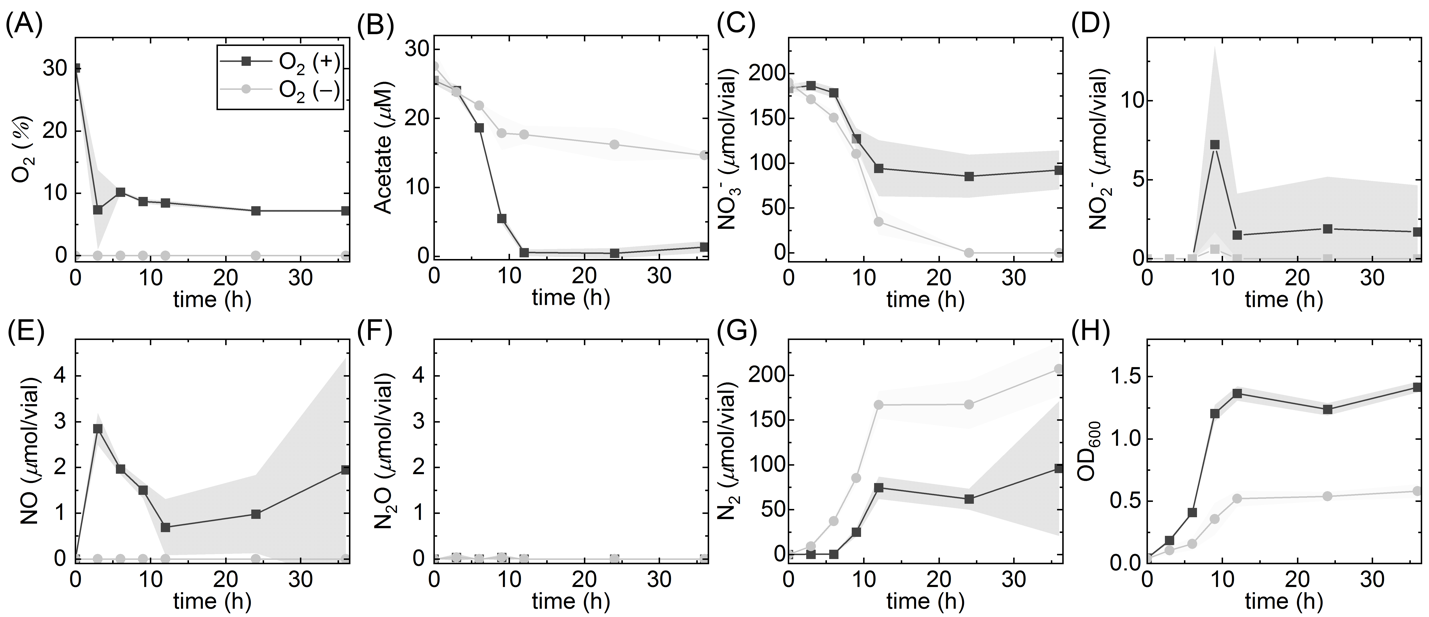
**

## **FIGURE S1**

## Concentrations of substrates, products, and cells for cultures of PD1222. Cultures were grown at 30°C in low oxic (dark grey data points) or anoxic (light grey data points) liquid batch cultures. Data are for (A) oxygen, (B) acetate, (C) nitrate, (D) nitrite, (E) nitric oxide, (F) nitrous oxide, (G) nitrogen gas, and (H) OD_600_ as a function of time. The data points are averages from three independent experimental replicates and the shaded regions are ± one standard deviation.

## **
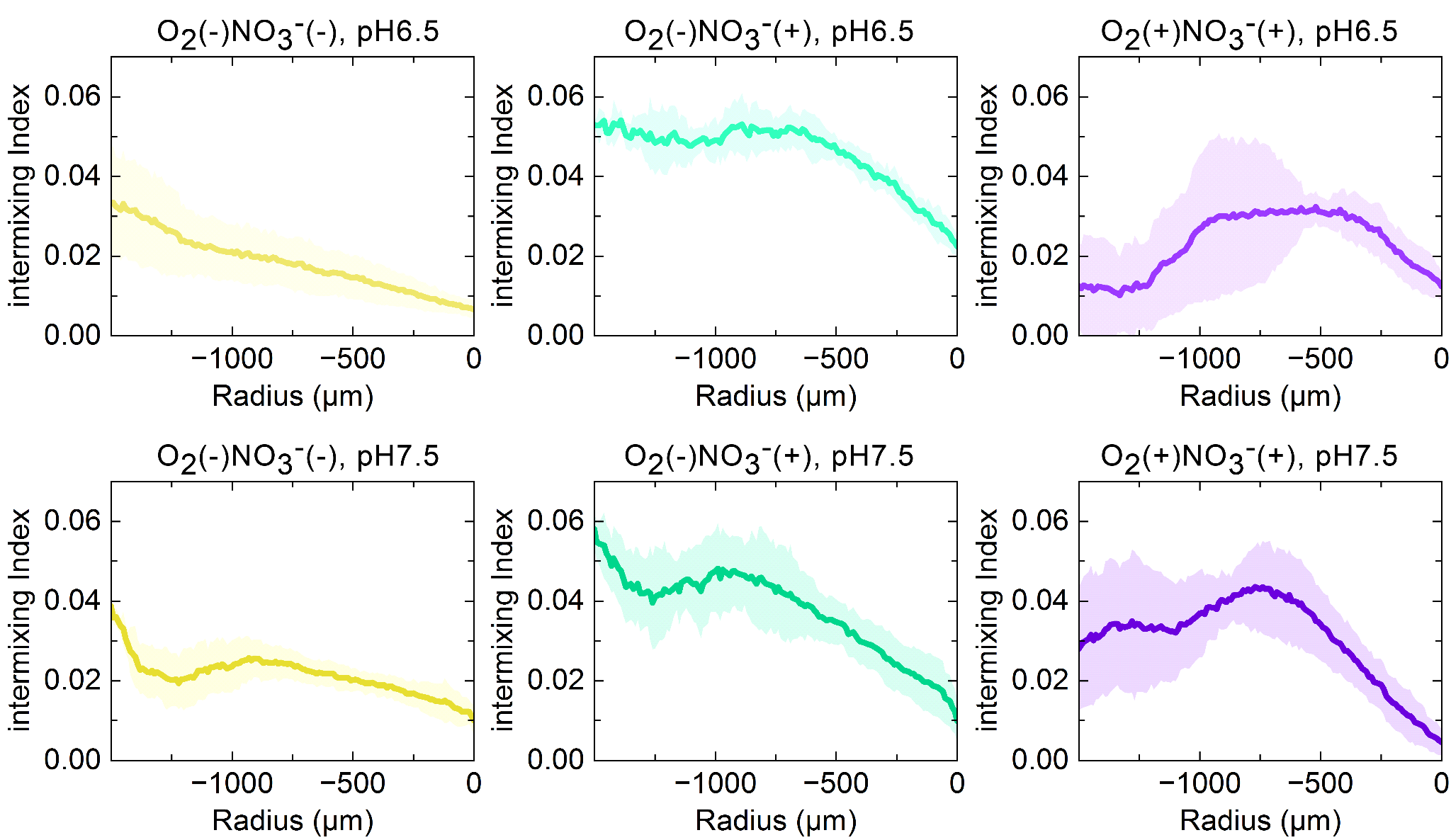
**

## **FIGURE S2**

## The intermixing index as a function of distance from the colony centroid. Mixtures of two sub-populations of PD1222 that express either red or green fluorescent protein were inoculated onto LB agar plates amended with or without 50 mM NO_3_^-^. The plates were then incubated at 30°C in an oxic or anoxic atmosphere for five days. The final colony periphery is defined as 0 radius and negative values indicate distances closer to the colony centroid. The data points are averages from three independent experimental replicates and the shaded regions are ± one standard deviation.

## **REFERENCES**

de Vries GE, Harms N, Hoogendijk J, Stouthamer AH. Isolation and characterization of Paracoccus denitrificans mutants with increased conjugation frequencies and pleiotropic loss of a (nGATCn) DNA-modifying property. Arch Microbiol 1989; 152: 52–57.

Simon R, Priefer U, Pühler A. A Broad Host Range Mobilization System for In Vivo Genetic Engineering: Transposon Mutagenesis in Gram Negative Bacteria. Biotechnology 1983; 1: 784–791.

Miller WG, Leveau JHJ, Lindow SE. Improved gfp and inaZ Broad-Host-Range Promoter-Probe Vectors. Mol Plant Microbe Interact 2000; 13: 1243–1250.

Morinaga K, Yamamoto T, Nomura N, Toyofuku M. Paracoccus denitrificans can utilize various long-chain N-acyl homoserine lactones and sequester them in membrane vesicles. Environ Microbiol Rep 2018; 10: 651–654.
